# Sex‐Specific Associations of Vascular Risk Factors With Abdominal Aortic Aneurysm: Findings From 1.5 Million Women and 0.8 Million Men in the United States and United Kingdom

**DOI:** 10.1161/JAHA.119.014748

**Published:** 2020-02-17

**Authors:** Jennifer L. Carter, Dylan R. Morris, Paul Sherliker, Rachel Clack, Kin Bong Hubert Lam, Alison Halliday, Robert Clarke, Sarah Lewington, Richard Bulbulia

**Affiliations:** ^1^ Clinical Trial Service Unit and Epidemiological Studies Unit Nuffield Department of Population Health University of Oxford United Kingdom; ^2^ MRC Population Health Research Unit Nuffield Department of Population Health University of Oxford United Kingdom; ^3^ Nuffield Department of Surgical Sciences University of Oxford United Kingdom; ^4^ Department of Vascular and Endovascular Surgery The Townsville Hospital Queensland Australia

**Keywords:** abdominal aortic aneurysm, risk factors, smoking, women, Cardiovascular Disease, Epidemiology, Risk Factors

## Abstract

**Background:**

Large studies are required for reliable estimates of important risk factors for abdominal aortic aneurysm (AAA). This could guide targeted AAA screening programs, particularly in subgroups like women who are currently excluded from such programs.

**Method and Results:**

In a cross‐sectional study, 1.5 million women and 0.8 million men without known vascular disease attended commercial screening clinics in the United Kingdom or United States from 2008 to 2013. Measurements of vascular risk factors were related to AAA using logistic regression with correction for regression dilution bias. Screening detected 12 729 new AAA cases (0.6%). Compared with never smoking, current smoking was associated with 15 times the risk of AAA among women (risk ratio 15.0, 95% CI 13.2–17.0) and 7 times among men (7.3, 6.4–8.2). In women aged <75 years, the risk of AAA was nearly 30 times greater in current smokers (26.4, 20.3–34.2). In every age group, the prevalence of AAA in female smokers was greater than in male never‐smokers. Positive log‐linear associations with AAA for women and men were also observed for usual body mass index, usual systolic blood pressure, height, usual low‐density lipoprotein cholesterol, and usual triglycerides.

**Conclusions:**

Log‐linear increases in the risks of AAA with traditional vascular risk factors should be considered when evaluating populations that may be at‐risk for the development of AAA, and when considering potential treatments. However, at any given age, female smokers are at higher risk of AAA than male never‐smokers, and a policy of screening male never‐smokers but not higher‐risk female smokers is questionable.

**Clinical Perspective**

**What Is New?**

- The importance of smoking as the principal risk factor for abdominal aortic aneurysm (AAA) in women has been underestimated substantially, with AAA risk being nearly 30 times greater in female smokers aged <75 years than in never‐smokers.
- At any given age, female smokers have double the risk of AAA than male never‐smokers.
- Log‐linear increases in the risks of AAA with other traditional vascular risk factors should be considered when evaluating at‐risk populations.

**What Are the Implications?**

- A policy of screening lower risk male never‐smokers for AAA, but not female current smokers, is questionable.

Abdominal aortic aneurysm (AAA) is an important cause of mortality in western countries, with a high fatality rate of ≈80% for ruptured AAAs contributing between 6000 and 8000 deaths each year in the United States and United Kingdom.^1^, ^2^ Many of these deaths are preventable, and the introduction of AAA screening programs for men in countries such as the United States and United Kingdom has been shown to reduce AAA‐related mortality.^3^ Currently, screening is only considered to be cost effective for men aged >65 years (United Kingdom), who additionally have a history of smoking (United States).^4^, ^5^

Previous research on risk factors for AAA have generally come from small studies where the role of chance could skew the magnitude of associations, particularly in subgroups with low prevalence of AAA.^6^, ^7^, ^8^ Furthermore, most reports have not excluded people with comorbid or pre‐existing cardiovascular disease.^7^ While associations with prior cardiovascular disease are important when estimating risk in screening populations, reverse causality arising from prior disease could have led to changes in modifiable risk factors such as smoking status, diet, body mass index (BMI), blood pressure and blood lipids, which would bias any observed associations.^7^ This study therefore aims to estimate the sex‐specific prevalence of screen‐detected AAA, and reliably assess among men and women the strength of the associations between vascular risk factors and AAA in a large cross‐sectional screening survey of 2.3 million adults (two‐thirds women) without prior cardiovascular disease or previously diagnosed AAA. Understanding the relative importance of risk factors for AAA could guide targeted AAA screening programs, particularly for women who are currently excluded from such programs. It may also provide insight into potentially effective treatments for AAA.

## Methods

### Screening Population

This was a cross‐sectional study of 1.5 million women and 0.8 million men who attended commercial screening clinics in the United States (98%) or United Kingdom between 2008 to 2012 and 2009 to 2013, respectively.^9^ Attendees self‐referred and self‐funded for a preventative health check that screened for risk factors that could lead to heart disease or stroke. Attendees underwent screening procedures that included: abdominal aortic aneurysm screening; carotid duplex screening; ankle‐brachial pressure index assessment; and a 12‐lead ECG. Attendees provided information on medical history and traditional vascular risk factors at the time of screening using a standardized questionnaire. A subset of attendees opted to have additional blood tests (see below). For the purposes of this study, analyses excluded those outside the age range of 35 to 89 years (0.4%), those with a prior history of atherosclerotic vascular disease (heart disease, stroke, and abdominal aortic aneurysm; 9.6%), those with missing AAA data (12.2%), and those with missing data or extreme values for risk factors (6.3%; Table S1). All attendees provided written consent for their de‐identified data to be analyzed for research purposes, and the study was approved by the University of Oxford Central University Research Ethics Committee. The authors had full access to the data in the study and take responsibility for their integrity and analysis. The data that support the findings of this study are available from the corresponding author upon reasonable request.

Demographic and self‐reported clinical history data were recorded for each attendee including age, sex, history of hypertension, history of diabetes mellitus (either diagnosis by a doctor, or report of hypoglycemic medications), smoking history (current smoker versus previous smoker versus never smoked), and cardiovascular medications (antiplatelet, antihypertensive, or lipid‐lowering drug). Weight and height were self‐reported in imperial units, and afterwards converted to SI units. BMI was calculated as weight (kg) /height (m^2^). Blood pressure was measured as part of an ankle‐brachial pressure index assessment. Standard blood pressure cuffs and sphygmomanometers were used, with systolic blood pressure (SBP) measured with a Doppler probe.

### Abdominal Aortic Aneurysm Screening

AAA screening was conducted by technicians using dedicated vascular ultrasound units (GE LOGIQ e, GE Healthcare, Waukesha, WI, USA). The maximum infra‐renal aortic diameter of the antero‐posterior and transverse planes was used to indicate aortic size. AAA was defined as a maximum infra‐renal aortic diameter ≥3 cm.

### Biochemical Measurements

A subset of attendees opted to have additional non‐fasting point‐of‐care blood tests, including lipid fractions (N=404 919 [17.4%]). Circulating total cholesterol, high density lipoprotein‐cholesterol (HDL‐C) and triglycerides were measured using enzymatic methods, and were quantified using the Alere Cholestech LDX system (Alere Inc, Waltham, MA, USA).^10^ Low density lipoprotein‐cholesterol (LDL‐C) was calculated using the Friedewald Formula (LDL‐C=total cholesterol–HDL‐C–[triglycerides/constant]).^11^

### Statistical Analysis

Prevalence was reported in terms of binomial proportions and 95% confidence limits, and stratified according to age group, sex, and smoking status. Multivariable logistic regression was used to assess the associations of vascular risk factors with AAA, overall and within sex‐specific subgroups; adjusted for age, country, and sex (when appropriate). Strictly this yields odds ratios, but as the prevalence of disease was low, these are almost identical to risk ratios (RRs), and they are described as RRs rather than odds ratios (for readability by non‐statisticians). The association of BMI with AAA was additionally adjusted for smoking status, as tobacco smoking and the diseases it causes may result in weight loss (reverse causation). Similarly, since lipid‐lowering therapies (in particular statins) markedly reduce circulating LDL‐C concentration and may inhibit the progression of vascular disease, the assessment of the associations between lipid fractions and AAA were restricted to people not taking lipid‐lowering drugs (310 512 [76.7% of subsample with lipid measurements]).^12^, ^13^ Similarly, analyses of SBP and AAA were limited to those not taking antihypertensive medication (1 460 570 [62.6% of full sample]), although sensitivity analyses checked associations in the total sample regardless of medication use for both lipids and blood pressure. Since some lipid fractions were moderately correlated, the association of lipid fractions with AAA were further adjusted for each other (eg, LDL‐C additionally adjusted for HDL‐C and triglycerides). Continuous risk factors were grouped into quantiles for plotting and to assess the shape of the relationship with AAA, and, if assessed to be approximately log‐linear, then used as continuous variables in analyses when estimating the strength of the associations. The comparative magnitude of vascular risk factors was assessed by standardizing the regression coefficients to one “usual” SD (see below). Because of the skewed distribution of triglycerides, estimates of triglycerides were conducted on the log scale and are presented as average risk ratios per 1.3 higher triglycerides levels (which corresponds to about 1 usual SD difference in log triglycerides levels).

Because of biological fluctuations and measurement error in a risk factor, using only a single measurement to classify the baseline exposure will cause the strength of any association to be underestimated (regression dilution bias).^14^, ^15^ Resurvey measurements were available for 10 245 attendees (0.43%) who underwent repeat screening at a median of 1.4 (IQR 1.2–2.4) years after baseline assessment, and can be used to correct for this underestimation in 2 ways (Table S2). First, for each continuous risk factor, risk ratios were estimated for groups defined by the baseline values, but plotted against the mean of the resurvey values (“usual value”). Second, self‐correlations between baseline and resurvey values were used to estimate the regression dilution ratio, and, for each risk factor, the standard deviation of the baseline measurements was multiplied by the regression dilution ratio to estimate the SD of the long‐term “usual” values.^14^ CIs in plots were calculated using the variance of the log risk in each group (including the reference group) which reflect the uncertainty in every group, including the reference group, and enables comparisons across exposure groups irrespective of the choice of a reference group.^16^, ^17^ All analyses were performed using SAS software version 9.3 (SAS Institute) and graphics were produced with the R statistical package, version 3.3.1 (www.r‐project.org/).

## Results

### Sample Characteristics

After exclusions for prior disease and missing data, 2.3 million of the original 3.3 million attendees remained (0.8 million men [35%] and 1.5 million women [65%]), with a mean (SD) age of 63.9 (10.0) years, BMI of 27.4 (4.6) kg/m^2^, and SBP of 132 (20) mm Hg (Table 1; Table S1). Nine percent of both male and female attendees were current smokers, 31% were taking lipid‐lowering medications and 37% were on antihypertensive medication. Of the 310 512 (13%) attendees with measurements of serum cholesterol that were not taking lipid‐lowering medication, the mean (SD) LDL‐C was 3.2 (0.9) mmol/L, HDL‐C was 1.4 (0.4) mmol/L and triglycerides was 1.3 (0.6) mmol/L.

**Table 1:** Characteristics of Screening Population

	Men	Women	All
	(818 616)	(1 513 327)	(2 331 943)
Characteristics			
AAA prevalence, %	9806 (1.2)	2923 (0.2)	12 729 (0.6)
Age, y	63.3±10.1	64.2±10.0	63.9±10.0
Height, m	1.8±0.1	1.6±0.1	1.7±0.1
Weight, kg	88.8±14.9	71.5±13.7	77.6±16.4
BMI, kg/m^2^	28.0±4.1	27.0±4.8	27.4±4.6
Systolic blood pressure, mm Hg	132±18	133±20	132±20
Smoking status, %^a^			
Current smoker	72 665 (9)	129 531 (9)	202 196 (9)
Ex‐smoker	256 026 (31)	359 698 (24)	615 724 (26)
Never‐smoker	410 006 (50)	870 301 (58)	1 280 307 (55)
Medical therapy, %^a^			
Lipid‐lowering	273 196 (33)	450 076 (30)	723 272 (31)
Antihypertensive(s)	309 070 (38)	562 303 (37)	871 373 (37)
Aspirin^b^	252 758 (31)	395 632 (26)	648 390 (28)
Lipids, mmol/L^c^			
LDL‐C	3.2±0.9	3.3±0.9	3.2±0.9
HDL‐C	1.2±0.4	1.5±0.4	1.4±0.4
Triglycerides^d^	1.3±0.6	1.2±0.6	1.3±0.6

Continuous variables presented as mean ± SD unless otherwise specified. Categorical variables presented as n (%). AAA indicates abdominal aortic aneurysm; BMI, body mass index; HDL‐C, high density lipoprotein‐cholesterol; LDL‐C, low density lipoprotein‐cholesterol.

a Based on participant report.

b History of aspirin therapy not collected in 20.38% of attendees.

c Lipids measured in subset of 310 512 attendees NOT taking medication for high cholesterol.

d Data presented as geometric mean±approximate SD.

### Association of Sociodemographic and Anthropometric Risk Factors With AAA

**Figure 1:**
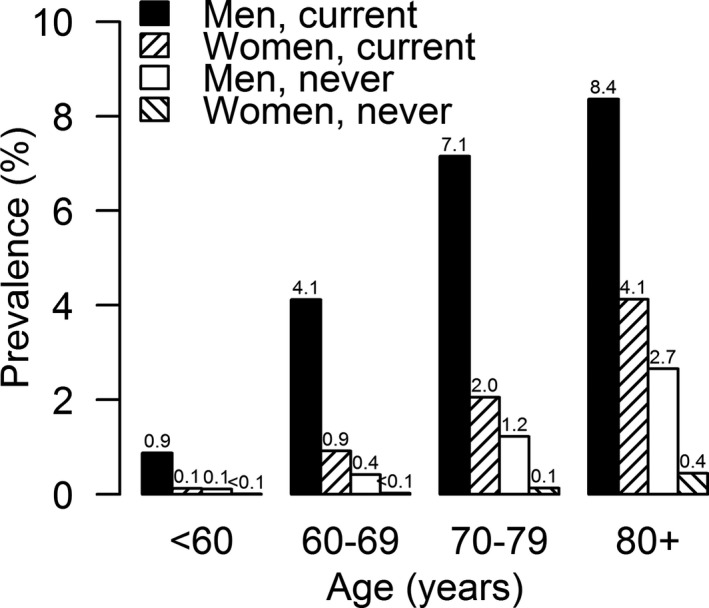
Prevalence of abdominal aortic aneurysm among 2 331 943 asymptomatic screenees, by age, sex, and smoking (current vs never).

The overall prevalence of AAA was 0.6% (95% CI: 0.54–0.56) (N=12 729). AAA was more common among men than women (age‐adjusted prevalence: 1.5% versus 0.25%), increasing with age in both (Figure 1). In each age group, the prevalence of AAA was substantially higher among smokers than non‐smokers. Among female smokers, the prevalence of AAA increased from 0.9% in those aged 60 to 69 years, to 4.1% in those aged >80 years and was higher than in never‐smoking men in every age group.

**Figure 2:**
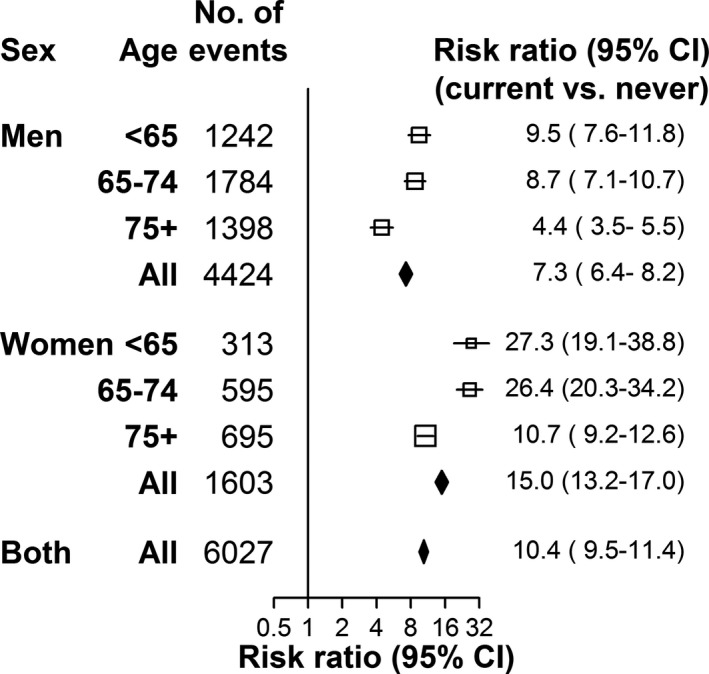
Associations of abdominal aortic aneurysm with current smoking, by age and sex. Risk ratios are adjusted for body mass index and country, sex, and age where appropriate.

Compared with never smokers, the risk of AAA among current smokers was ≈7 times greater for men (RR 7.3, 95% CI: 6.4–8.2), but 15 times greater in women (RR 15.0, 13.2–17.0) (Figure 2). The associations were particularly strong in women aged <75 years, with the risk of AAA among current smokers almost 30 times that of never smokers (26.4, 20.3–34.2).

**Figure 3:**
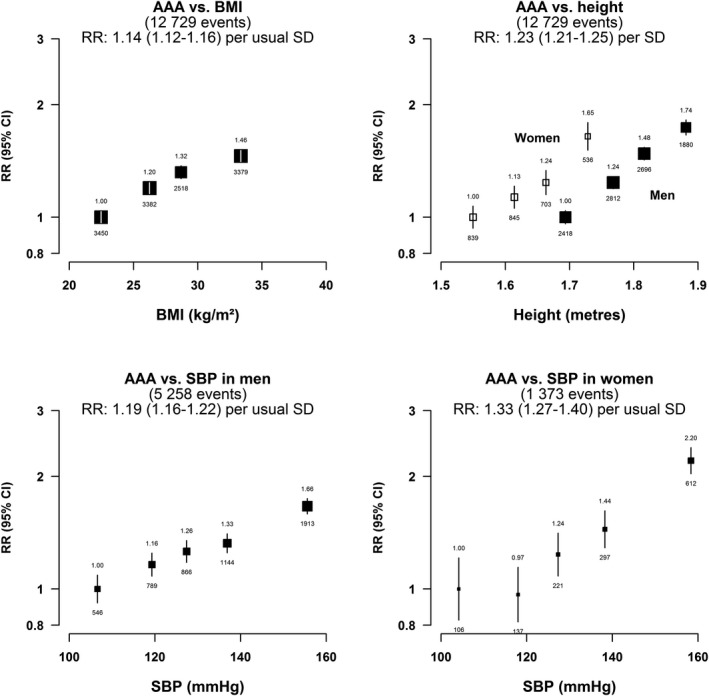
Associations of abdominal aortic aneurysm with usual systolic blood pressure, BMI, and height in men and women. Risk ratios are adjusted for age, sex, and country, and are plotted against the means of the resurvey values. BMI is additionally adjusted for smoking. Usual SD: BMI=4.0 kg/m^2^; systolic blood pressure=12.9 mm Hg; height (men)=0.07 m; height (women)=0.07 m. AAA indicates abdominal aortic aneurysm; BMI, body mass index; RR, risk ratio; SBP, systolic blood pressure.

Positive log‐linear associations between physical measurements and AAA are shown in Figure 3. Each additional 4.0 kg/m^2^ higher BMI (ie, 1 usual SD) was associated with a 14% higher risk of AAA (RR 1.14; 95% CI 1.12–1.16), and this was similar in men and women (Table 2, p_heterogeneity_=0.20). Each 12.9 mmHg higher usual SBP was associated with a 22% (1.22; 1.19–1.25) higher risk; and this was somewhat stronger in women (1.33, 1.27–1.40) than men (1.19, 1.19–1.25) (p_het_=0.0001). For height, each 7 cm increase was associated with about a 23% higher risk of AAA in both men (1.23, 1.21–1.26) and women (1.22, 1.17–1.26).

**Table 2:** Associations of continuous vascular risk factors with abdominal aortic aneurysm by sex. Risk ratios per 1 usual SD are reported

Risk Factor	Women		Men		Overall		Heterogeneity
	Usual SD	RR (95% CI)	Usual SD	RR (95% CI)	Usual SD	RR (95% CI)	
Height, cm	0.07	1.22 (1.17–1.26)	0.07	1.23 (1.21–1.26)	0.10	1.23 (1.21–1.26)	0.60
SBP, mm Hg	13.2	1.33 (1.27–1.40)	12.2	1.19 (1.16–1.22)	12.9	1.22 (1.19–1.25)	0.0001
BMI, kg/m^2^	4.3	1.17 (1.13–1.21)	3.6	1.14 (1.11–1.16)	4.0	1.14 (1.12–1.16)	0.20
Lipids, mmol/L							
LDL‐C	0.66	1.30 (1.16–1.46)	0.64	1.16 (1.09–1.24)	0.65	1.19 (1.13–1.26)	0.09
HDL‐C (lower)	0.36	1.40 (1.22–1.62)	0.31	1.37 (1.27–1.48)	0.37	1.38 (1.29–1.47)	0.76
Log triglycerides^a^	1.29	1.01 (0.87–1.16)	1.30	1.14 (1.06–1.22)	1.29	1.11 (1.04–1.18)	0.13

Overall risks ratios calculated as the inverse‐variance average of the women and men sex‐specific associations. BMI, body mass index; HDL‐C, high density lipoprotein‐cholesterol; LDL‐C, low density lipoprotein‐cholesterol; RR, risk ratio; SBP, systolic blood pressure.

a Usual SD=exp(logSD×regression dilution ratio).

### Association of Biochemical Measurements With AAA

**Figure 4:**
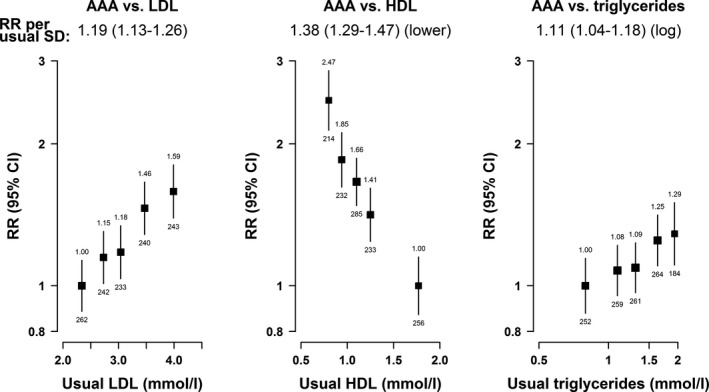
Associations of abdominal aortic aneurysm with lipid fractions. Risk ratios are adjusted for age, sex, country, and other lipid fractions, and are plotted against the means of the resurvey values. Usual SD: Low density lipoprotein‐cholesterol=0.65 mmol/L; high density lipoprotein‐cholesterol=0.37 mmol/L; triglycerides=1.3‐fold higher. AAA indicates abdominal aortic aneurysm; LDL, low‐density lipoprotein; HDL, high‐density lipoprotein; RR, risk ratio.

Log‐linear relationships were observed for each of the blood lipids with AAA, independent of each other, with positive associations for usual LDL‐C and usual triglycerides and an inverse association for usual HDL‐C (Figure 4). The risk of AAA was 19% higher (RR 1.19; 95% CI 1.13–1.26) per 0.65 mmol/L higher usual LDL‐C and 11% (1.11; 1.04–1.18) per 1.3‐fold higher usual triglycerides. Of all the continuous risk factors analyzed, the strongest log‐linear association was seen between HDL‐C and AAA, with a 0.37 mmol/L lower usual HDL‐C associated with a 38% higher risk of AAA (1.38; 1.29–1.47). These associations were similar for men and women (Table 2).

In sensitivity analyses for lipids and SBP that did not exclude participants taking lipid‐lowering or antihypertensive treatments, results were marginally weaker (eg, LDL‐C: 1.19 [1.13–1.26] excluding treatment versus 1.10 [1.05–1.16] no exclusions; SBP: 1.22 [1.19–1.25] versus 1.18 [1.16–1.20]).

## Discussion

To our knowledge, this is one of the largest studies of abdominal aortic aneurysm, where 1.5 million women and 0.8 million men without prior disease attended a commercial screening clinic in the United States and United Kingdom, and 12 729 screened positive for AAA. Among women, the risk of screen‐detected AAA was 15 times higher among current smokers compared with never smokers and was 7 times higher among men. Among women aged <75 years, the risks were nearly 30 times greater for current smokers than for never‐smokers.

The relative risks reported in this study for smoking contrast with 2 previous meta‐analyses on risk factors for AAA which reported that current smoking increased the risk of AAA by only 2 to 3 times.^6^, ^7^ The much stronger associations shown here may be attributable to the exclusion of participants with prior cardiovascular disease, since inclusion of participants with pre‐existing disease in previous studies may have underestimated the association if participants had recently quit smoking because of their diagnosis (reverse causality). The extremely high relative risks of AAA for women smokers in this study are particularly important since small AAA rupture is more likely in women, and continued smoking increases the rate of AAA growth and doubles the risk of rupture.^18^ Importantly, for determining screening strategies, the prevalence of AAA in female smokers in this study was higher than in never‐smoking men in every age group examined. This finding is consistent with a smaller prospective study which reported that the lifetime risk of AAA for female smokers was similar to that in men who were ex‐smokers and greater than that in men who were never smokers.^19^ Previous evaluations of AAA screening in women were too underpowered to make any recommendations,^4^ and hence women are currently not included in United States and United Kingdom AAA screening programs. However, these findings suggest that larger studies could have the power to reliably evaluate the cost‐effectiveness of potential screening programs in important subgroups, such as women who smoke.

Few modifiable risk factors for AAA, other than smoking, have previously been identified. An important strength of this study was its ability to demonstrate reliably strong associations with AAA across a range of modifiable vascular risk factors for both women and men; including BMI, SBP, LDL‐C and triglycerides (all positive) and HDL‐C (inverse). Our results also show that these associations were continuous across the range of values recorded, with no thresholds above or below which the associations plateaued. Previous research has been equivocal, reporting weak or null associations for these risk factors and few studies have had the power to examine these associations separately for men and women.^7^, ^8^, ^18^, ^19^, ^20^, ^21^, ^22^, ^23^ Furthermore, previous studies have not corrected for regression dilution bias, and so the strength of the associations would have been weakened by biological fluctuations and measurement error in the risk factors.^15^

Prior research on the association of lipids with AAA has been complicated by examination of different lipid measurements, the lack of adjustment for lipid‐lowering medication and the correlation between different lipoproteins.^8^, ^24^ Analyses on total cholesterol and LDL‐C have been mixed, with meta‐analyses of observational studies showing no association with AAA for LDL‐C;^24^, ^25^, ^26^, ^27^, ^28^ although observational evidence on the inverse association with HDL‐C has been more consistent.^24^, ^26^, ^27^, ^28^ After excluding for lipid‐lowering therapies and adjusting for correlations amongst lipid fractions in the current study, there were positive log‐linear associations for LDL‐C and triglycerides, and an inverse association for HDL‐C that was the strongest association apart from smoking. Similarly, a meta‐analysis of Mendelian Randomization studies of lipids and AAA concluded that there were positive associations with LDL‐C and triglycerides, and an inverse association with HDL‐C; the effect sizes in that meta‐analysis were similar to this study for HDL‐C but were stronger for LDL‐C and triglycerides.^29^

Even though women were on average shorter than men (suggesting they would have smaller aortas), positive log‐linear associations between height and AAA were still apparent across the range of values for both men and women, with similar effect sizes. While other research has documented similar positive associations between height and AAA, a previous meta‐analysis of prospective data reported inverse associations for height with coronary disease, stroke, and heart failure.^30^ The positive associations with height for both sexes in the current study, as well as with the traditional vascular risk factors, support the idea that while the development of AAA may share many vascular risk factors with other forms of cardiovascular disease, the etiology of AAA may also be due to some distinct processes.

The extremely large size of the sample recruited across the commercial screening centers is a key strength of this study, ensuring reliable analyses with a substantial number of participants measured across the range of each risk factor and in important subgroups like women. In particular, the inclusion of biochemical measurements allowed investigations with AAA that have not previously been studied on such a large scale. Furthermore, resurvey measurements enabled the associations to be corrected for regression dilution bias, which allows the true strength of the associations to be reported. Although efforts were made to avoid reverse causality by excluding participants with prior cardiovascular disease and medication, these measures were self‐reported and so there would inevitably have been some reporting errors which could potentially bias risk ratios in either direction. There is also likely to have been some residual confounding because of confounding factors measured with error and unmeasured confounders. Since this study was cross‐sectional, causality cannot be inferred for any of the associations observed. Finally, participants were self‐referred for screening, so the sample in this study was not representative. Although this means that the prevalence of AAA is probably underestimated, associations with risk factors will still be valid as they are not affected by selection bias.^31^

## Conclusions

Smoking is a particularly strong risk factor for AAA among men and women at all ages, but for women aged <75 years it is associated with nearly a 30‐fold higher risk of AAA. Consequently, whilst female smokers are at lower absolute risk of AAA than male smokers, at any given age female smokers are at higher risk of AAA than male never‐smokers. A policy of screening low‐risk male never‐smokers but not higher risk female smokers is therefore questionable. Log‐linear increases in the risks of AAA were also reported across both sexes for height, SBP, BMI, LDL cholesterol, and triglycerides, with inverse associations documented for HDL cholesterol. These characteristics should be considered when evaluating populations that may be at‐risk for the development of AAA, and when considering treatments (medical and non‐medical) that may be effective for managing the natural progression of this potentially fatal disease.

## Sources of Funding

This work was supported by core funding to the Medical Research Council‐Population Health Research Unit. The Clinical Trial Service Unit and Epidemiological Studies Unit also receives direct support from the British Heart Foundation and Cancer Research UK. Dr Morris is supported by a General Sir John Monash Scholarship and an Avant Doctor in Training Research Scholarship. Prof Lewington is supported by the Medical Research Council of the UK and received a Goodger and Schorstein Scholarship from the University of Oxford Medical Sciences Division for this project. Funders had no role in the study design, data collection, data analysis, manuscript preparation, or publication decisions. Authors had complete access to the study data that support the publication.

## Disclosures

None.

## Supporting information


**Table S1.** Number of Participants and Reasons for Exclusion
**Table S2.** Calculation of Regression Dilution Ratios

## References

[1] Stather PW , Sidloff DA , Rhema IA , Choke E , Bown MJ , Sayers RD . A review of current reporting of abdominal aortic aneurysm mortality and prevalence in the literature. Eur J Vasc Endovasc Surg. 2014;47:240–242. 24368205 10.1016/j.ejvs.2013.11.007

[2] Reimerink JJ , van der Laan MJ , Koelemay MJ , Balm R , Legemate DA . Systematic review and meta‐analysis of population‐based mortality from ruptured abdominal aortic aneurysm. Br J Surg. 2013;100:1405–1413. 24037558 10.1002/bjs.9235

[3] Guirguis‐Blake JM , Beil TL , Sun X , Senger CA , Whitlock EP . Primary care screening for abdominal aortic aneurysm: a systematic evidence review for the U.S. Preventive Services Task Force [Internet]. Rockville, MD: Agency for Healthcare Research and Quality (US); 2014 Jan. (Evidence Syntheses, No. 109.) 4, Discussion. Available from: https://www.ncbi.nlm.nih.gov/books/NBK184795/. Accessed March 4, 2019. 24555205

[4] Cosford PA , Leng GC , Thomas J . Screening for abdominal aortic aneurysm. Cochrane Database Syst Rev. 2007;2 10.1002/14651858.CD002945.pub2. 17443519

[5] LeFevre ML . Screening for abdominal aortic aneurysm: US Preventive Services Task Force recommendation statement. Ann Intern Med. 2014;161:281–290. 24957320 10.7326/M14-1204

[6] Altobelli E , Rapacchietta L , Profeta V , Fagnano R . Risk factors for abdominal aortic aneurysm in population‐based studies: a systematic review and meta‐analysis. Int J Environ Res Public Health. 2018;15:2805. 10.3390/ijerph15122805 PMC6313801 30544688

[7] Cornuz J , Sidoti Pinto C , Tevaearai H , Egger M . Risk factors for asymptomatic abdominal aortic aneurysm: systematic review and meta‐analysis of population‐based screening studies. Eur J Public Health. 2004;14:343–349. 15542867 10.1093/eurpub/14.4.343

[8] Nordon IM , Hinchliffe RJ , Loftus IM , Thompson MM . Pathophysiology and epidemiology of abdominal aortic aneurysms. Nat Rev Cardiol. 2011;8:92. 21079638 10.1038/nrcardio.2010.180

[9] Savji N , Rockman CB , Skolnick AH , Guo Y , Adelman MA , Riles T , Berger JS . Association between advanced age and vascular disease in different arterial territories: a population database of over 3.6 million subjects. J Am Coll Cardiol. 2013;61:1736–1743. 23500290 10.1016/j.jacc.2013.01.054

[10] Allain CC , Poon LS , Chan CS , Richmond W , Fu PC . Enzymatic determination of total serum cholesterol. Clin Chem. 1974;20:470–475. 4818200

[11] Friedewald WT , Levy RI , Fredrickson DS . Estimation of the concentration of low‐density lipoprotein cholesterol in plasma, without use of the preparative ultracentrifuge. Clin Chem. 1972;18:499–502. 4337382

[12] Kang S , Wu Y , Li X . Effects of statin therapy on the progression of carotid atherosclerosis: a systematic review and meta‐analysis. Atherosclerosis. 2004;177:433–442. 15530920 10.1016/j.atherosclerosis.2004.08.005

[13] van Lammeren GW , den Ruijter HM , Vrijenhoek JE , van der Laan SW , Velema E , de Vries JP , de Kleijn DP , Vink A , de Borst GJ , Moll FL , Bots ML , Pasterkamp G . Time‐dependent changes in atherosclerotic plaque composition in patients undergoing carotid surgery. Circulation. 2014;129:2269–2276. 24637558 10.1161/CIRCULATIONAHA.113.007603

[14] MacMahon S , Peto R , Cutler J , Collins R , Sorlie P , Neaton J , Abbott R , Godwin J , Dyer A , Stamler J . Blood pressure, stroke, and coronary heart disease. Part 1, Prolonged differences in blood pressure: prospective observational studies corrected for the regression dilution bias. Lancet. 1990;335:765–774. 1969518 10.1016/0140-6736(90)90878-9

[15] Clarke R , Shipley M , Lewington S , Youngman L , Collins R , Marmot M , Peto R . Underestimation of risk associations due to regression dilution in long‐term follow‐up of prospective studies. Am J Epidemiol. 1999;150:341–353. 10453810 10.1093/oxfordjournals.aje.a010013

[16] Easton DF , Peto J , Babiker AG . Floating absolute risk: an alternative to relative risk in survival and case‐control analysis avoiding an arbitrary reference group. Stat Med. 1991;10:1025–1035. 1652152 10.1002/sim.4780100703

[17] Plummer M . Improved estimates of floating absolute risk. Stat Med. 2004;23:93–104. 14695642 10.1002/sim.1485

[18] Sweeting M , Thompson S , Brown L , Powell J . Meta‐analysis of individual patient data to examine factors affecting growth and rupture of small abdominal aortic aneurysms. Br J Surg. 2012;99:655–665. 22389113 10.1002/bjs.8707

[19] Tang W , Yao L , Roetker NS , Alonso A , Lutsey PL , Steenson CC , Lederle FA , Hunter DW , Bengtson LG , Guan W . Lifetime risk and risk factors for abdominal aortic aneurysm in a 24‐year prospective study: the ARIC study (atherosclerosis risk in communities). Arterioscler Thromb Vasc Biol. 2016;36:2468–2477. 27834688 10.1161/ATVBAHA.116.308147 PMC5397388

[20] Howard D , Banerjee A , Fairhead J , Handa A , Silver L , Rothwell P , Study OV . Age‐specific incidence, risk factors and outcome of acute abdominal aortic aneurysms in a defined population. Br J Surg. 2015;102:907–915. 25955556 10.1002/bjs.9838 PMC4687424

[21] Lederle FA , Johnson GR , Wilson SE , Chute EP , Littooy FN , Bandyk D , Krupski WC , Barone GW , Acher CW , Ballard DJ . Prevalence and associations of abdominal aortic aneurysm detected through screening. Ann Intern Med. 1997;126:441–449. 9072929 10.7326/0003-4819-126-6-199703150-00004

[22] Lee A , Fowkes F , Carson M , Leng G , Allan P . Smoking, atherosclerosis and risk of abdominal aortic aneurysm. Eur Heart J. 1997;18:671–676. 9129900 10.1093/oxfordjournals.eurheartj.a015314

[23] Stackelberg O , Björck M , Sadr‐Azodi O , Larsson S , Orsini N , Wolk A . Obesity and abdominal aortic aneurysm. Br J Surg. 2013;100:360–366. 23203847 10.1002/bjs.8983

[24] Golledge J , Van Bockxmeer F , Jamrozik K , McCann M , Norman PE . Association between serum lipoproteins and abdominal aortic aneurysm. Am J Cardiol. 2010;105:1480–1484. 20451699 10.1016/j.amjcard.2009.12.076

[25] Chun KC , Teng KY , Chavez LA , Van Spyk EN , Samadzadeh KM , Carson JG , Lee ES . Risk factors associated with the diagnosis of abdominal aortic aneurysm in patients screened at a regional veterans affairs health care system. Ann Vasc Surg. 2014;28:87–92. 24189004 10.1016/j.avsg.2013.06.016

[26] Forsdahl SH , Singh K , Solberg S , Jacobsen BK . Risk factors for abdominal aortic aneurysms. Circulation. 2009;119:2202–2208. 19364978 10.1161/CIRCULATIONAHA.108.817619

[27] Takagi H , Manabe H , Umemoto T . A meta‐analysis of association between serum lipoproteins and abdominal aortic aneurysm. Am J Cardiol. 2010;106:753–754. 10.1016/j.amjcard.2010.06.021 20723658

[28] Stather PW , Sidloff DA , Dattani N , Gokani VJ , Choke E , Sayers RD , Bown MJ . Meta‐analysis and meta‐regression analysis of biomarkers for abdominal aortic aneurysm. Br J Surg. 2014;101:1358–1372. 25131707 10.1002/bjs.9593

[29] Weng L‐C , Roetker NS , Lutsey PL , Alonso A , Guan W , Pankow JS , Folsom AR , Steffen LM , Pankratz N , Tang W . Evaluation of the relationship between plasma lipids and abdominal aortic aneurysm: a Mendelian randomization study. PLoS One. 2018;13:e0195719. 29649275 10.1371/journal.pone.0195719 PMC5896990

[30] Collaboration ERF . Adult height and the risk of cause‐specific death and vascular morbidity in 1 million people: individual participant meta‐analysis. Int J Epidemiol. 2012;41:1419–1433. 22825588 10.1093/ije/dys086 PMC3465767

[31] Fry A , Littlejohns TJ , Sudlow C , Doherty N , Adamska L , Sprosen T , Collins R , Allen NE . Comparison of sociodemographic and health‐related characteristics of UK Biobank participants with those of the general population. Am J Epidemiol. 2017;186:1026–1034. 28641372 10.1093/aje/kwx246 PMC5860371

