# Correction to: Sex‐Specific Associations of Vascular Risk Factors With Abdominal Aortic Aneurysm: Findings From 1.5 Million Women and 0.8 Million Men in the United States and United Kingdom

**DOI:** 10.1161/JAHA.119.014559

**Published:** 2020-07-09

## Correction

In the article by Carter et al, “Sex‐Specific Associations of Vascular Risk Factors With Abdominal Aortic Aneurysm: Findings From 1.5 Million Women and 0.8 Million Men in the United States and United Kingdom,” which published online on February 17, 2020, and appeared in the February 18, 2020 issue of the journal, (J Am Heart Assoc. 2020;9:e014748. DOI: 10.1161/JAHA.119.014748.), a funding source was omitted.

The Sources of Funding statement has been corrected to include:

“Professor Halliday's research is funded by the UK Health Research (NIHR) Oxford Biomedical Research Centre (BRC).”

The authors regret the error.

The online version of the article has been updated and is available at https://www.ahajo​urnals.org/doi/10.1161/JAHA.119.014748

