# Supplementary material for: Sex‐Specific Associations of Vascular Risk Factors With Abdominal Aortic Aneurysm: Findings From 1.5 Million Women and 0.8 Million Men in the United States and United Kingdom
Source: J Am Heart Assoc. 2020 Feb 17;9(4):e014748. doi: 10.1161/JAHA.119.014748 (PMC7070225; doi:10.1161/JAHA.119.014748)
Supplement: Supplementary file 1 — Table S1. Number of Participants and Reasons for Exclusion Table S2. Calculation of Regression Dilution Ratios [file JAH3-9-e014748-s001.pdf]

SUPPLEMENTAL MATERIAL

Table S1. Number of participants and reasons for exclusion.

Reason Excluded	Number of Participants	(%)	Number remaining	% remaining
**Total Attendees***	**3 258 027**	**100**	**3 258 027**	**100**
**Reason for Exclusion****				
Age < 35	12 341	0.4	3 245 686	99.6
Prior Disease (AAA, CHD, Stroke)	311 200	9.6	2 934 486	90.1
Missing AAA	396 346	12.2	2 538 140	77.9
Missing BMI or BMI <15 kg/m^2 or ≥40 kg/m^2	176 418	5.4	2 361 722	72.5
Missing SBP or SBP <80 mmHg or SBP >240 mmHg	23 263	0.7	2 338 459	71.8
Missing Height or Height <1.4m or Height >2.0 m	6 516	0.2	2 331 943	71.6
**Total in Analyses**			**2 331 943**	**71.6**

AAA, abdominal aortic aneurysm; CHD, coronary heart disease; BMI, body mass index; SBP, systolic blood pressure

*after those with missing date of birth/sex or aged <18 or 90 or over excluded
**sequential exclusion

Table S2. Calculation of regression dilution ratios.

Continuous Variable	Regression Dilution Ratio
BMI	0·88*
SBP	0·68
HDL-C	0·84
LDL-C	0·73
logTriglycerides	0·56

Resurvey measurements were available for 10 324 attendees (0.43%) who underwent repeat screening at a median of 1·4 [IQR 1·2-2·4] years after baseline assessment. Regression dilution ratios calculated as correlation of baseline measurements with resurvey measurements. Not calculated for height as self-reported values are consistent over time. SBP, systolic blood pressure; BMI, body mass index; LDL-C, low density lipoprotein-cholesterol; HDL-C, high density lipoprotein-cholesterol

*Calculated from self-reported weight and height.
